# Chemistry of polyhalogenated nitrobutadienes, 14: Efficient synthesis of functionalized (*Z*)-2-allylidenethiazolidin-4-ones

**DOI:** 10.3762/bjoc.10.170

**Published:** 2014-07-17

**Authors:** Viktor A Zapol’skii, Jan C Namyslo, Mimoza Gjikaj, Dieter E Kaufmann

**Affiliations:** 1 Institute of Organic Chemistry, Clausthal University of Technology, Leibnizstr. 6, 38678 Clausthal-Zellerfeld, Germany; 2 Institute of Inorganic and Analytical Chemistry, Clausthal University of Technology, Paul-Ernst-Str. 4, 38678 Clausthal-Zellerfeld, Germany

**Keywords:** atropisomers, cyclization, 2-nitroperchlorobutadiene, 1*H*-pyrazoles, thiazolidin-4-ones

## Abstract

The reaction of mercaptoacetic acid esters with pentachloro-2-nitro-1,3-butadiene (**1**) provides an appropriate precursor for the synthesis of special thiazolidin-4-ones. Applying different anilines as the second constituent for the requisite cyclization step, a series of (*Z*)-2-allylidenethiazolidin-4-ones was obtained in yields up to 81%. Some subsequent reactions have been examined too, such as the formation of perfunctionalized 1*H*-pyrazoles upon treatment with hydrazine. Thiazolidinones are as well known for their physiological activities as for their application in optoelectronics.

## Introduction

Preliminary studies in the field of polyhalogenated nitrobutadienes have already shown the enormous potential of pentachloro-2-nitro-1,3-butadiene (**1**) as a precursor for the “click synthesis” of highly functionalized (hetero)cyclic as well as acyclic compounds [1–15]. The corresponding syntheses that we have developed up to now [2–13], always start with the attack of an appropriate nucleophile at the activated terminal carbon atom of the nitrodichlorovinyl group within **1** to undergo a vinylic substitution. Thus, in case of, e.g., sulfur nucleophiles, the corresponding thioperchlorobutadiene derivatives are easily accessible [2,5,16–17]. In this paper, we describe the formation of uniquely substituted thiazolidin-4-ones, a class of compounds which has proven to exhibit distinctive bioactivity, e.g., antifungal, antibacterial, antitubercular, and anticonvulsant properties [18–28].

Additionally, more recent studies often focus on their anticancer [29–31] and anti-HIV potential [30,32]. Several 4-thiazolidinone derivatives such as ralitoline (anticonvulsant), etozoline (antihypertensive), pioglitazone (hypoglycemic) and thiazolidomycin (activity against streptomyces species) are already in the market [33]. Beyond that, among industrial applications thiazolidinones serve as electron donors, e.g., as ligands in coordination compounds or as vulcanizing agents [31]. Furthermore, it is well known that these heterocycles play a major role in corresponding organic electronics, e.g., they lead to high quantum efficiency as donor compound in solar cells [34]. Very recently, thiazolidinones became a target in the field of photonics and optoelectronics, potentially applicable for reversible optical data storage, photo switching of optical elements, photochromic polymers, and similar applications [35].

The background of thiazolidinone chemistry including classical synthetic strategies has been reviewed comprehensively [18–28,36–39]. Even though among possible synthetic pathways the use of mercaptoacetic acid and its derivatives is well-established [40–44], our approach, starting from a mercaptoacetic acid derivative of perchloro-2-nitro-1,3-butadiene and an aniline, is unprecedented up to now. In this context, it is very interesting that the reaction of a 2-nitro-1-thioperchlorobuta-1,3-diene with N-nucleophiles usually yields a 1-amino-2-nitro-1-thioperchlorobuta-1,3-diene [16,45]. However, our novel ring closing reaction incorporates an arylamine as source for the nitrogen of the thiazolidin-4-one heterocycle.

## Results and Discussion

**Scheme 1:**
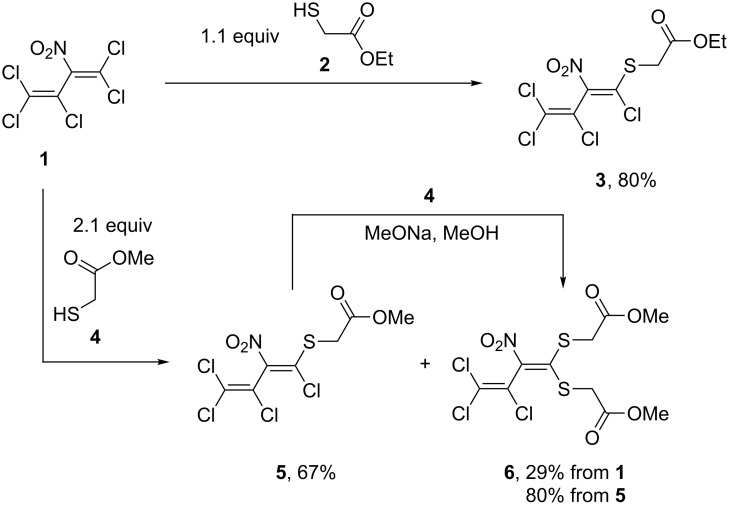
S_N_Vin reactions of pentachloro-2-nitro-1,3-butadiene (**1**).

The reaction of pentachloro-2-nitro-1,3-butadiene (**1**) with 1.1 equivalents of ethyl 2-mercaptoacetate (**2**) at room temperature without a solvent for 10 days furnished ethyl 2-((1,3,4,4-tetrachloro-2-nitrobuta-1,3-dien-1-yl)thio)acetate (**3**) as a single isomer in 80% yield (for further details see Supporting Information File 1). In contrast, the conversion of **1** (neat) with 2.1 equivalents of methyl 2-mercaptoacetate (**4**) results in a mixture of methyl 2-((1,3,4,4-tetrachloro-2-nitrobuta-1,3-dien-1-yl)thio)acetate (**5**) and 1,1-bis(methoxycarbonylmethylthio)-2-nitro-3,4,4-trichlorobuta-1,3-diene (**6**) after 10 days at room temperature with 67% and 29% yields, respectively. These nitrodienes **5** and **6** were separated by flash column chromatography and then characterized. Interestingly, the dithio compound **6** was also accessible by treatment of the aforementioned monothio derivative **5** with one equivalent of mercaptoacetate **4**. Following this pathway, the disubstituted diene **6** was obtained in 80% yield (Scheme 1).

**Scheme 2:**
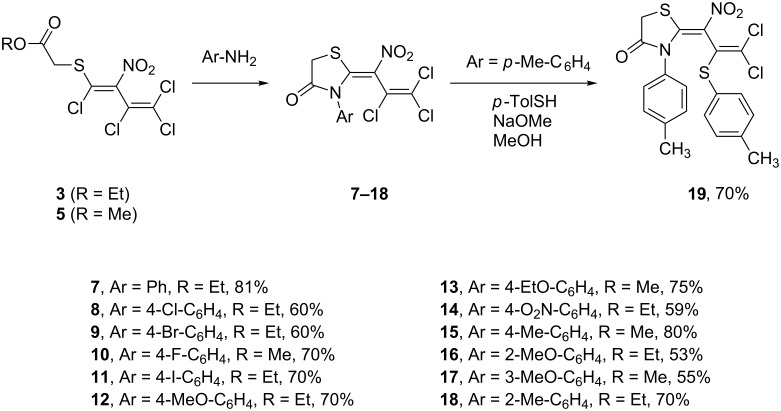
Formation of thiazolidin-4-ones **7–19**.

The subsequent reaction of the mercaptoacetates **3** and **5** with 2.1 equivalents of various arylamines is the key step of our thiazolidin-4-one synthesis, that led to the corresponding (*Z*)-3-aryl-2-(2,3,3-trichloro-1-nitroallylidene)thiazolidin-4-ones **7**–**18** with up to 81% yield. As an example for the sequential reactivity of the persubstituted allylidene side chain, the thiazolidin-4-one **15** was reacted with *p*-tolylthiolate. Thereby, the C(2)–Cl position of the allylidene group was selectively substituted by the reactive thiolate nucleophile, again in a S_N_Vin-type process, to give (*Z*)-3-(4-tolyl)-2-(3,3-dichloro-1-nitro-2-[4-tolylthio]allylidene)thiazolidin-4-one (**19**) in 70% yield (Scheme 2).

**Figure 1:**
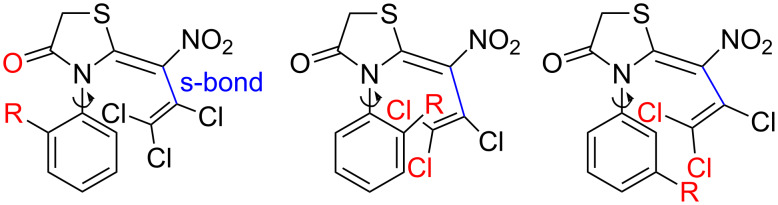
Hindered rotation in the case of *ortho*- or *meta*-substituted aniline precursors.

According to proton NMR, the ring-closing step with the parent aniline or with one of its *para*-substituted derivatives in each case led to a single *N*-arylthiazolidin-4-one isomer **7**–**15**. In the case of the *ortho*- or *meta*-substituted anilines **16**–**18** two atropisomers were generated. This phenomenon is due to a less hindered rotation of the C–N bond of the former anilines (Figure 1).

**Figure 2:**
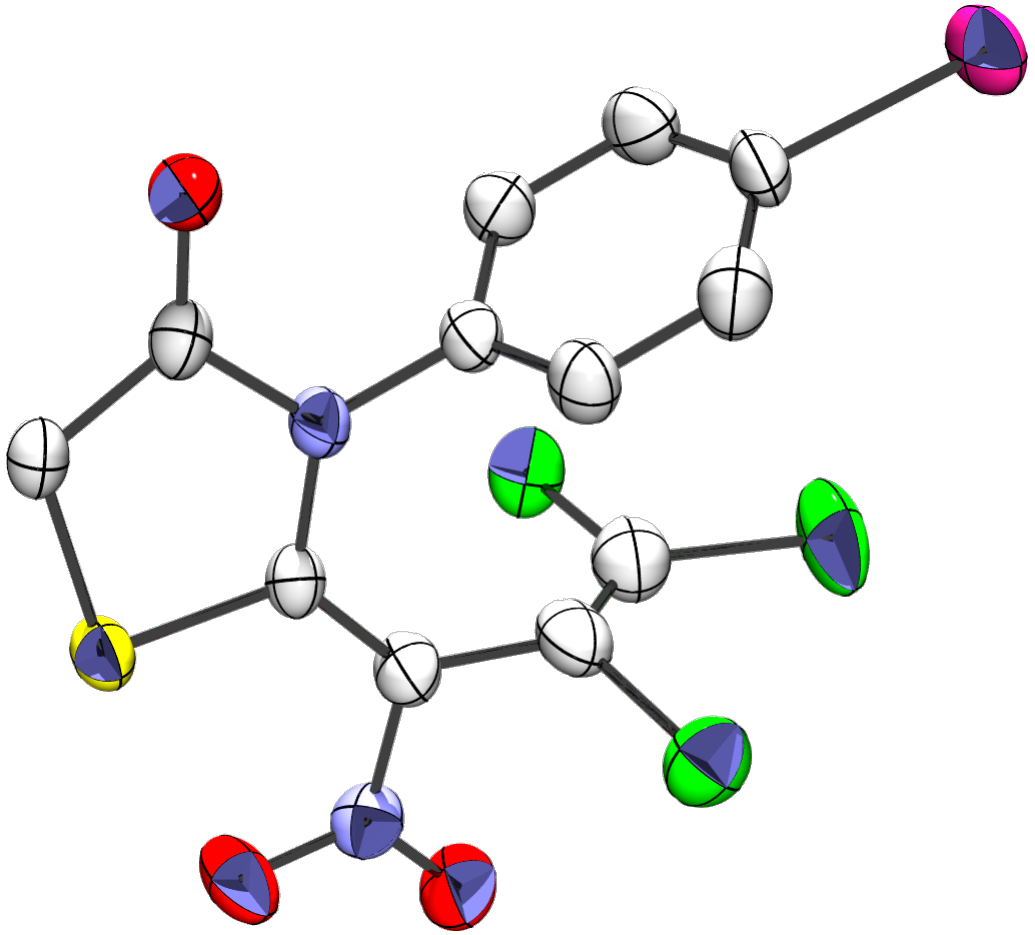
X-ray analysis of thiazolidin-4-one **11**.

The steric hindrance is additionally forcing by the s-*cis* conformation of the nitrobutadiene moiety (cf. Figure 2). A DFT calculation of the energy barrier for rotation of the aromatic substituent resulted in extraordinary high values of 169 and 191 kJ/mol for the *ortho*-methyl compound **18** and the larger, but less rigid *ortho*-methoxy derivative **16**, respectively. As expected, the *meta*-anisyl derivative **17** showed a lower barrier of 73 kJ/mol (see Supporting Information File 1). For comparison, the well-known atropisomer 1,1’-binaphthalene-2,2’-diol (BINOL) shows a rotational barrier of 158 kJ/mol with calculated data in good accordance with the experimental value [46].

In addition to substantial NMR, IR, and mass spectral characterization of the thiazolidinones **7**–**18**, as an example the 4-iodophenyl substituted thiazolidinone **11** was subjected to X-ray analysis (see Supporting Information File 1). This confirmed the *Z*-configuration of the inner double bond which bears the sulfur atom of the hetero ring as well as the nitro group. The structural plot also gives an idea of the abovementioned bulkiness, even in the case of the depicted *para*-substitution (Figure 2).

**Scheme 3:**
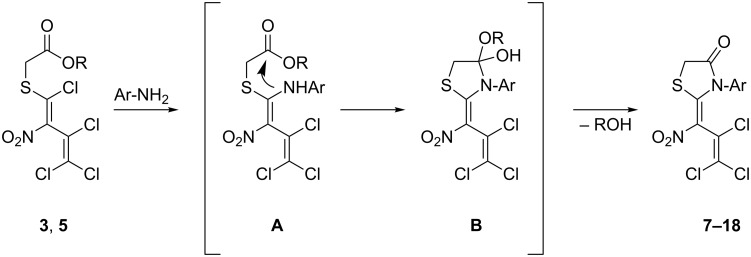
Assumed mechanism for the formation of thiazolidin-4-ones **7–18**.

The formation of the thiazolidin-4-ones **7**–**18** is assumed to consist of three individual steps. In a S_N_Vin type reaction with the aniline derivative intermediate **A** (Scheme 3) is formed. Subsequently, the thus introduced amino group attacks the carbonyl carbon of the mercaptoacetate moiety. This ring closure affords the temporary imide hemiacetal **B** which is then stabilized upon elimination of the corresponding alcohol to give the desired thiazolidin-4-ones **7**–**18** (Scheme 3).

**Scheme 4:**
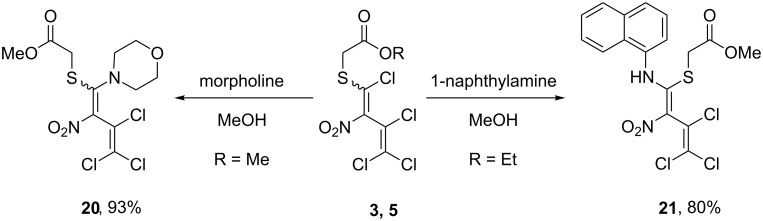
Substitution reactions of the precursors **3** and **5** with additional amines.

Furthermore, in addition to the anilines described above (Scheme 2), we applied 1-naphthylamine as a bulkier aromatic representative and morpholine as a secondary, more basic aliphatic amine. Conversion of **3** and **5** with morpholine and 1-naphthylamine afforded the open chain products **20** and **21** in 93% and 80% yield, respectively (Scheme 4), without a subsequent cyclization under one-pot conditions. Unexpectedly, even under forcing conditions cyclization of isolated **21** did not take place. Compound **21** was exclusively obtained as *E*-isomer. This was indicated by a proton NMR signal shifted downfield to about 12 ppm due to a strong hydrogen bond between the amino hydrogen and the nitro group. In contrast, lacking the opportunity to build such a hydrogen bridge, the regiochemistry of compound **20** (also formed as one single isomer) remained unclear.

With the thiazolidinones in hand, in the case of **7**, **8**, **10**, and **12** we carried out a Knoevenagel-type condensation with benzaldehyde and 3,4-dichlorobenzaldehyde in acetic acid at 118 °C in the presence of triethylamine. Thus, the 5-arylmethylidenethiazolidin-4-ones **22**–**26** were obtained in 68–94% yield. The presence of only one signal for the benzylidene proton at 7.83–7.98 ppm in the ^1^H NMR spectra of **22**–**26** suggested the formation of a single isomer, which was assigned to the *Z*-configuration according to the literature for similar compounds [47–48]. Interestingly, close analogues of these structures, i.e., the 5-arylmethylidene rhodanines, possess photosynthesis-inhibiting and antialgal properties [49], show anticancer activity [50–51], and are inhibitors of bacterial enzyme synthetase MurD with *E. coli* [52].

**Scheme 5:**
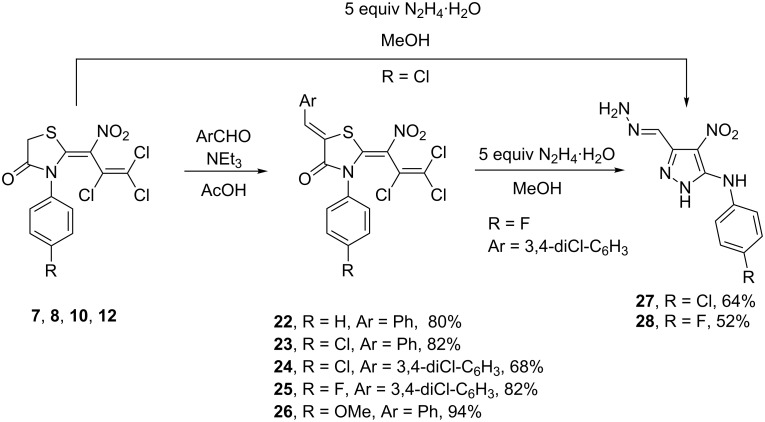
Synthesis of 5-arylmethylidenethiazolidin-4-ones **22**–**26** and 1*H*-pyrazoles **27**, **28**.

Additional treatment of thiazolidin-4-one **8** with a fivefold excess of hydrazine hydrate resulted in the formation of a 1*H*-pyrazole: A total of two hydrazine molecules were incorporated into the final product, whereas two additional equivalents were consumed capturing hydrochloric acid. In this way, the 1*H*-pyrazole **27** with an exceptional substitution pattern was obtained in 64% yield. The same reaction was applied to 5-arylmethylidene-thiazolidin-4-one **25** which furnished the corresponding 1*H*-pyrazole **28** in 52% yield (Scheme 5).

**Scheme 6:**
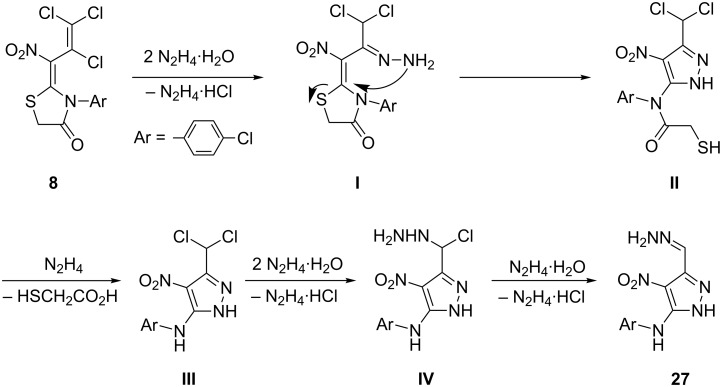
Assumed mechanism for the formation of 1*H*-pyrazole **27**.

A plausible multistep mechanism of this conversion is shown in Scheme 6: Initially, a first molecule of the strong nucleophile hydrazine is assumed to substitute the single C–Cl group within the trichlorovinyl subunit of **8** to give the intermediate **I** (S_N_Vin reaction). Thereby, a second equivalent of hydrazine captures hydrochloric acid. The amino group of the hydrazone then attacks the electrophilic C-2 position of the thiazolidinone. This way a threefold substituted 1*H*-pyrazole is formed (intermediate **II**) under ring opening of the thiazolidinone. In the course of the reaction, probably at this stage, the *N*-aryl amide is hydrazinolysed to give the intermediate **III**, which is converted into the monohydrazino compound **IV** upon consumption of two further equivalents of hydrazine. Finally, **27** is obtained upon elimination of hydrochloric acid, caught by a fifth equivalent of hydrazine (Scheme 6). An analogous mechanism is also plausible for the formation of pyrazole **28** from thiazolidinone **25**. However, instead of the elimination of 2-mercaptoacetic acid from intermediate **II**, in the latter case 3-(3,4-dichlorophenyl)-2-mercaptoacrylic acid is released.

**Scheme 7:**
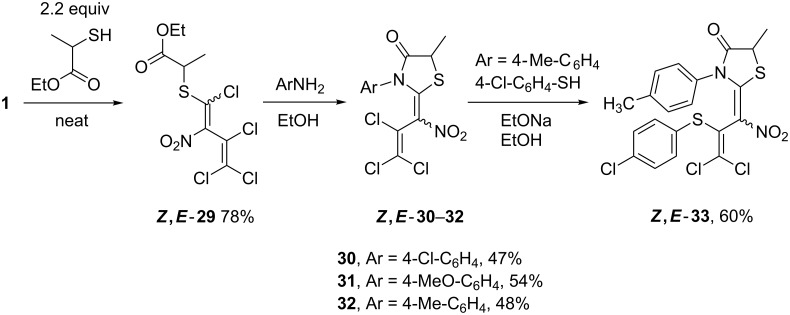
Formation of ethyl propanoate **29** and subsequent reactions.

Moreover, nitrodiene **1** was subjected to a vinylic substitution with ethyl 2-mercaptopropanoate to give ethyl 2-[(1,3,4,4-tetrachloro-2-nitrobuta-1,3-dien-1-yl)thio]propanoate (**29**) as an inseparable mixture of *Z*- and *E*-isomers in a 4:1 ratio with 78% total yield. Treatment of dienes (*Z,E*)-**29** with different anilines in ethanol at 0 °C to rt furnished (*Z,E*)-3-aryl-5-methylthiazolidinones **30**–**32**. As an example, thiazolidinone **32** was reacted with 4-chlorothiophenol in the presence of sodium ethoxide to give 4-chlorophenylthio compound **33** (60%), again as a mixture of *Z*- and *E*-isomers, but this time in a 2:1 ratio (Scheme 7).

## Conclusion

A two-step synthesis of 2-allylidene-*N*-arylthiazolidinones **7**–**18** has been developed, starting from our building block 2-nitroperchlorobutadiene (**1**), 2-mercaptoacetates **2** and **4**, and anilines. Inclusion of *o*- or *m*-substituted *N*-aryl groups leads to the formation of stable thiazolidinone atropisomers, due to hindered rotation between the trichlorovinyl and the arylamino groups. X-ray analysis proved the *Z*-configuration of the nitrovinylidene group of **11**. Subsequent reactions of the thiazolidinones, such as the S_N_Vin thiolation in 2-position of the allylidene backbone, Knoevenagel condensation of the heterocyclic rings with aromatic aldehydes, and an unusual formation of persubstituted pyrazoles demonstrate the chemical versatility of these heterocyclic systems. The newly formed thiazolidinones deserve additional synthetic interest as starting materials, also due to their trichlorovinyl group. Tests on their physiological activities and potential applications as optoelectronic materials are on the way.

## Supporting Information

File 1 Experimental part.
