# Supplementary material for: Chemistry of polyhalogenated nitrobutadienes, 14: Efficient synthesis of functionalized (Z)-2-allylidenethiazolidin-4-ones
Source: Beilstein J Org Chem. 2014 Jul 17;10:1638–44. doi: 10.3762/bjoc.10.170 (PMC4142838; doi:10.3762/bjoc.10.170)
Supplement: File 1 — Experimental part. [file Beilstein_J_Org_Chem-10-1638-s001.pdf]

Supporting Information

for

Chemistry of polyhalogenated nitrobutadienes, 14: Efficient synthesis of functionalized (*Z*)-2-allylidenethiazolidin-4-ones

Viktor A. Zapol'skii[1], Jan C. Namyslo[1], Mimoza Gjikaj[2] and Dieter E. Kaufmann*[1]

Address: [1]Institute of Organic Chemistry, Clausthal University of Technology, Leibnizstr. 6, 38678 Clausthal-Zellerfeld, Germany and [2]Institute of Inorganic and Analytical Chemistry, Clausthal University of Technology, Paul-Ernst-Str. 4, 38678 Clausthal-Zellerfeld, Germany

Email: Dieter E. Kaufmann* - dieter.kaufmann@tu-clausthal.de

*Corresponding author

Experimental part

Melting points were measured on a Büchi 520 apparatus and were uncorrected. NMR spectra were obtained on a Bruker Avance with 400 MHz proton frequency. ^{1}H NMR spectra in $CDCl_3$ were referenced to TMS as an internal standard. ^{13}C NMR spectra refer to the solvent signal center at 77.0 ppm ($CDCl_3$). In case of DMSO-d_6, the solvent peak was set to 2.50 ppm (^{1}H) and 39.70 ppm (^{13}C), respectively. ^{14}N NMR spectra were obtained in 1D-mode and were externally referenced to nitromethane at 0.0 ppm. ^{15}N NMR spectra were recorded as two-dimensional ^{1}H,^{15}N HMBC with gradient selection using the same reference. IR spectra were obtained on a Bruker 'Vector 22' FTIR as film between NaCl plates or as KBr pellets. Mass spectra were recorded on a Hewlett Packard 'MS 5989B' with direct inlet. Standard and high-resolution mass spectra were measured with a Bruker Daltonik 'APEX IV' 7 T fourier transform ion cyclotron resonance mass spectrometer with electrospray ionisation at the Institute of Organic Chemistry, University of Göttingen, Germany.

In the case of chlorinated and brominated compounds, all peak values of molecular ions as well as fragments refer to the isotopes ^{35}Cl and ^{79}Br. TLC analyses were carried out on Merck plates coated with silica gel (60 F 254). Silica gel 60 was also used for column chromatography.

All density-functional theory (DFT) calculations were carried out by using the Jaguar 7.7.107 software (Schrödinger, München, Germany) running on Linux 2.6.18-238.el5 SMP (x86_64) on two AMD Phenom II X6 1090T processor workstations (Beowulf-cluster) parallelized with OpenMPI 1.3.4. MM2 optimized structures were used as starting geometries. Complete geometry optimizations were carried out on the implemented LACVP* (Hay-Wadt effective core potential (ECP) basis on heavy atoms, N31G6* for all other atoms) basis set and with the B3LYP density functional. All calculated structures were proven to be true minima by the absence of imaginary frequencies. Plots were obtained using Maestro 9.1.207, the graphical interface of Jaguar. Rotational barriers have been calculated fully relaxed, fixating one torsion angle around the rotated bond, and optimizing all remaining degrees of freedom. Torsion angles were modified in steps of 10°.

Pentachloro-2-nitro-1,3-butadiene (**1**) was prepared from 2*H*-pentachloro-1,3-butadiene in 53% yield (b.p. 69–71 °C, 1 mbar) according to the literature [1].

Ethyl 2-((1,3,4,4-tetrachloro-2-nitrobuta-1,3-dien-1-yl)thio)acetate (**3**): Neat nitrobutadiene **1** (27.13 g, 0.10 mol) was charged with ethyl 2-mercaptoacetate (**2**) (13.22 g, 0.11 mol) and stirred for 10 d at room temperature (rt). The resulting precipitate was filtered off and washed twice with 20 mL portions of cold petroleum ether. The crude product **3** (yellow solid, 28.40 g, 80%) was found to be pure enough for the next step without further purification. M.p. 55–56 °C. IR (KBr): $\tilde{\nu}$ 2991, 2934, 1731 (C=O), 1528 (NO_2), 1366, 1291 (NO_2), 1205, 1169, 1006, 925, 886, 831, 758, 685 cm^{-1}. ^1H NMR ($CDCl_3$): δ 4.47 (q, J = 7.1 Hz, 2 H, OCH_2), 3.91 (s, 2 H, SCH_2), 1.28 (t, J = 7.1 Hz, 3 H, CH_3) ppm. ^{13}C NMR ($CDCl_3$): δ 166.5 (CO), 156.4 (Cl-C-S), 138.9 (CNO_2), 128.8 (CCl), 120.7 (CCl_2), 62.4 (OCH_2), 37.3 (SCH_2), 13.8 (CH_3) ppm. ^{14}N NMR ($CDCl_3$): δ -14.8 ppm. MS (EI, 70 eV): *m/z* (%) = 353 (5) [M^+], 318 (10), 266 (100), 203 (47), 188 (27). HRMS (ESI): calcd. for $C_8H_8Cl_4NO_4S$ $[M + H]^+$ 353.89227; found 353.89227.

Methyl 2-(1,3,4,4-tetrachloro-2-nitrobuta-1,3-dienylthio)acetate (**5**) **and dimethyl 2,2'-(3,4,4-trichloro-2-nitrobuta-1,3-diene-1,1-diyl)bis(sulfanediyl)diacetate (6):** A solution of 2-mercapto-acetate **4** (2.23 g, 21 mmol) in nitrobutadiene **1** (2.71 g, 10 mmol) was stirred for 10 d at rt. After addition of water (100 mL) the mixture was extracted with dichloromethane (3 × 50 mL). The combined organic layers were washed with water (2 × 50 mL) and dried with anhydrous $CaCl_2$. Removal of the solvent and subsequent flash column chromatography (diethyl ether/petroleum ether 1:1) afforded acetate **5** (2.31 g, 67%) and butadiene **6** (1.21 g, 29%).

Methyl 2-(1,3,4,4-tetrachloro-2-nitrobuta-1,3-dienylthio)acetate (5). R_f (diethyl ether/petroleum ether 3:1) = 0.62; m.p. 39–41 °C; yellow solid. IR (KBr): $\tilde{\nu}$ 3001, 2955, 1743 (C=O), 1538 (NO_2), 1386, 1295 (NO_2), 1202, 1157, 1009, 926, 888, 828, 760, 687 cm^{-1}. ^1H NMR ($CDCl_3$): δ 3.95 (s, 2 H, SCH_2), 3.82 (s, 3 H, OCH_3) ppm. ^{13}C NMR ($CDCl_3$): δ 167.2 (CO), 155.9 (Cl-C-S), 139.4 (CNO_2), 129.2 (CCl), 120.8 (CCl_2), 53.3 (OCH_3), 37.2 (SCH_2) ppm. MS (EI, 70 eV): *m/z* (%) = 339 (5) [M^+), 304, (12), 266 (100), 203 (72), 188 (44). HRMS (ESI): calcd. for $C_7H_6Cl_4NO_4S$ [M + H]$^+$: 339.87662; found: 339.87687.

Dimethyl 2,2'-(3,4,4-trichloro-2-nitrobuta-1,3-diene-1,1-diyl)bis(sulfanediyl)diacetate (6). R_f (diethyl ether/petroleum ether 3:1) = 0.48; yellow oil. IR (film): $\tilde{\nu}$ 2951, 1740 (C=O), 1601, 1534 (NO_2), 1436, 1384, 1299 (NO_2), 1198, 1164, 999, 936, 886, 824, 762, 683 cm^{-1}. ^1H NMR ($CDCl_3$): δ 3.95 (s, 2 H, SCH_2), 3.78 (s, 6 H, OCH_3), 3.76 (s, 2 H, SCH_2) ppm. ^{13}C NMR ($CDCl_3$): δ 168.3 (CO), 167.9 (CO), 155.1 (S-C-S), 140.1 (CNO_2), 129.0 (CCl), 121.6 (CCl_2), 53.2 (2 OCH_3), 37.9 (SCH_2), 37.1 (SCH_2) ppm. MS (EI, 70 eV): *m/z* (%) = 409 (3) [M^+], 374 (6), 336 (100), 270 (59), 258 (32). HRMS (ESI): calcd. for $C_{10}H_{11}Cl_3NO_6S_2$ [M + H]$^+$: 409.90879; found: 409.90895.

Synthesis of butadiene **6** from acetate **5**. At 0 °C sodium methoxide (0.054 g, 1.00 mmol) was added to a suspension of acetate **5** (0.34 g, 1.00 mmol) in anhydrous methanol (5 mL) portionwise within 5 min. At first, the resulting mixture was stirred for 1 h at 0 °C, then the cooling bath was removed. After 15 h at rt, water (50 mL) and 3 drops of conc. hydrochloric acid were added. Extraction with dichloromethane (3 × 15 mL), treatment of the combined organic layers twice with water (10 mL each portion), and drying with anhydrous calcium chloride gave a pure product that was subsequently dried in vacuo to yield butadiene **6** (0.33 g, 0.8 mmol, 80%).

General procedure for the preparation of the thiazolidinones 7–18 and 30–32.
At 0 °C a solution of the appropriate aromatic amine (22.0 mmol) in ethanol, methanol, or THF (50 mL) was added dropwise within 10 min to a suspension of the acetate **3**, **5** or **29** (10.0 mmol) in the same solvent (50 mL). After stirring for 2 h at 0 °C the mixture was allowed to warm to rt. Subsequent to additional stirring for 2 d, the resulting precipitate was filtered off and consecutively washed with ethanol (1 × 20 mL), 5% HCl (2 × 50 mL), water (2 × 50 mL), and again ethanol (1 × 30 mL).

(*Z*)-3-Phenyl-2-(2,3,3-trichloro-1-nitroallylidene)thiazolidin-4-one (7). Yield: 81%, m.p. 205–206 °C; pink solid. IR (KBr): $\tilde{\nu}$ 2987, 2922, 1754 (CO), 1597, 1526 (NO_2), 1392, 1363, 1288 (NO_2), 1178, 960, 927, 911, 881, 814, 757, 702, 687, 660, 561 cm^{-1}. ^1H NMR (DMSO-d_6): δ 7.63–7.29 (m, 5 H, Ph), 4.19 (d, J = 18.7 Hz, 1 H, SCH_2), 4.09 (d, J = 18.7 Hz, 1 H, SCH_2) ppm. ^{13}C NMR (DMSO-d_6): δ

174.1 (CO), 165.8 (NCS), 134.5 (NC_q), 130.5 (CH), 129.5 (CH), 129.1 (CH), 128.9 (CH), 128.6 (CCl), 127.5 (CH), 121.4, 121.3, 32.6 (SCH_2) ppm. MS (EI, 70 eV): *m/z* (%) = 364 (4) [M^+], 328 (10), 264 (11), 254 (11), 236 (27), 219 (63), 210 (17). HRMS (ESI): calcd. for $C_{12}H_8Cl_3N_2O_3S$ $[M + H]^+$: 364.93157; found: 364.93167.

(*Z*)-3-(4-Chlorophenyl)-2-(2,3,3-trichloro-1-nitroallylidene)thiazolidin-4-one (8). Yield: 60%, m.p. 223–225 °C; yellow solid. IR (KBr): $\tilde{\nu}$ 2976, 2936, 1738 (CO), 1604, 1518 (NO_2), 1489, 1365, 1292 (NO_2), 1178, 1091, 967, 920, 883, 822, 795, 725, 682, 584, 501, 463 cm^{-1}. ^1H NMR (DMSO-d_6): δ 7.65–7.56 (m, 2 H, Aryl), 7.52-7.43 (m, 2 H, Aryl), 4.18 (d, *J* = 18.7 Hz, 1 H, SCH_2), 4.09 (d, *J* = 18.7 Hz, 1 H, SCH_2) ppm. ^{13}C NMR (DMSO-d_6): δ 173.9 (CO), 165.7 (NCS), 135.3 (NC_q), 133.4 ($C_{q,aryl}Cl$), 130.9 (CH), 129.6 (CH), 129.5 (CH), 129.2 (CH), 128.7 (CCl), 121.4 (CCl_2), 121.2 (CNO_2), 32.7 (SCH_2) ppm. MS (EI, 70 eV): *m/z* (%) = 398 (5) [M^+], 362 (14), 297 (28), 269 (38), 252 (100), 245 (20), 210 (17). HRMS (ESI): calcd. for $C_{12}H_7Cl_4N_2O_3S$ $[M + H]^+$: 398.89260; found: 398.89248.

(*Z*)-3-(4-Bromophenyl)-2-(2,3,3-trichloro-1-nitroallylidene)thiazolidin-4-one (9). Yield: 60%, m.p. 230–232 °C; dark-yellow solid. IR (KBr): $\tilde{\nu}$ 2975, 2935, 1736 (CO), 1602, 1519 (NO_2), 1486, 1364, 1290 (NO_2), 1173, 1069, 965, 918, 882, 818, 794, 760, 716, 696, 575, 493 cm^{-1}. ^1H NMR (DMSO-d_6): δ 7.79–7.69 (m, 2 H, Aryl), 7.44–7.34 (m, 2 H, Aryl), 4.20 (d, *J* = 18.7 Hz, 1 H, SCH_2), 4.10 (d, *J* = 18.7 Hz, 1 H, SCH_2) ppm. ^{13}C NMR (DMSO-d_6): δ 173.9 (CO), 165.6 (NCS), 133.8 (NC_q), 132.4 (CH), 132.1 (CH), 131.1 (CH), 129.7 (CH), 128.9 (CCl), 124.0 (CBr), 121.4 (CCl_2), 121.2 (CNO_2), 32.7 (SCH_2) ppm. MS (EI, 70 eV): *m/z* (%) = 442 (4) [M^+], 406 (17), 362 (4), 315 (30), 298 (92), 287 (17), 254 (100). HRMS (ESI): calcd. for $C_{12}H_7BrCl_3N_2O_3S$ $[M + H]^+$: 442.84208; found: 442.84208.

(*Z*)-3-(4-Fluorophenyl)-2-(2,3,3-trichloro-1-nitroallylidene)thiazolidin-4-one (10). Yield: 70%, m.p. 218–219 °C; yellow solid. IR (KBr): $\tilde{\nu}$ 2976, 2936, 1744 (CO), 1605, 1521 (NO_2), 1461, 1367, 1288 (NO_2), 1176, 967, 920, 883, 832, 760, 714, 687, 613, 540, 498 cm^{-1}. ^1H NMR (DMSO-d_6): δ 7.57–7.31 (m, 4 H, Aryl), 4.18 (d, *J* = 18.7 Hz, 1 H, SCH_2), 4.09 (d, *J* = 18.7 Hz, 1 H, SCH_2) ppm. ^{13}C NMR (DMSO-d_6): δ 174.1 (CO), 165.8 (NCS), 162.8 (CF, $J_{C,F}$ = 247.3 Hz), 131.6 (CH, $J_{C,F}$ = 9.6 Hz), 130.9 ($NC_{q,}$ $J_{C,F}$ = 3.1 Hz), 130.3 (CH, $J_{C,F}$ = 9.6 Hz), 128.6 (CCl), 121.4 (CCl_2), 121.2 (CNO_2), 116.6 (CH, $J_{C,F}$ = 23.0 Hz), 116.4 (CH, $J_{C,F}$ = 23.4 Hz), 32.6 (SCH_2) ppm. MS (EI, 70 eV): *m/z* (%) = 346 (5) $[M – HCl]^+$, 313 (2), 253 (9), 237 (28), 192 (24). HRMS (ESI): calcd. for $C_{12}H_7Cl_3FN_2O_3S$ $[M + H]^+$: 382.92215; found: 382.92214.

(*Z*)-3-(4-Iodophenyl)-2-(2,3,3-trichloro-1-nitroallylidene)thiazolidin-4-one (11). Yield: 70%, m.p. 244–246 °C; orange solid. IR (KBr): $\tilde{\nu}$ 2976, 2934, 1737 (CO), 1602, 1519 (NO_2), 1482, 1364, 1291 (NO_2), 1175, 1009, 965, 919, 883, 833, 760, 712, 694, 573, 492 cm^{-1}. ^1H NMR (DMSO-d_6): δ 7.95–

7.83 (m, 2 *H*, Aryl), 7.28–7.16 (m, 2 H, Aryl), 4.18 (d, J = 18.7 Hz, 1 H, SCH_2), 4.08 (d, J = 18.7 Hz, 1 H, SCH_2) ppm. ^{13}C NMR (DMSO-d_6): δ 174.1 (CO), 165.8 (NCS), 162.8 (CF, $J_{C,F}$ = 247.3 Hz), 131.6 (CH, $J_{C,F}$ = 9.6 Hz), 130.9 (NC_q, $J_{C,F}$ = 3.1 Hz), 130.3 (CH, $J_{C,F}$ = 9.6 Hz), 128.6 (CCl), 121.4 (CCl_2), 121.2 (CNO_2), 116.6 (CH, $J_{C,F}$ = 23.0 Hz), 116.4 (CH, $J_{C,F}$ = 23.4 Hz), 32.6 (SCH_2) ppm. MS (EI, 70 eV): *m/z* (%) = 490 (3) [M^+], 455 (21), 361 18), 344 (100), 299 (48), 287 (12). HRMS (ESI): calcd. for $C_{12}H_7Cl_3IN_2O_3S$ $[M + H]^+$: 490.82822; found: 490.82828.

X-ray crystallographic study. $C_{12}H_6Cl_3IN_2O_3S$: The compound crystallizes in the monoclinic crystal system: space group $P2_1/c$ (No. 14) with a = 17.425(1), b = 8.8144(6), c = 10.704(2) Å, β = 102.38(1)º, V = 1605.7(3) $Å^3$, Z = 4, R1 = 0.0756, wR2 = 0.2061.

The X-ray diffraction data were collected on a STOE IPDS diffractometer with Mo K_α radiation. The structures were dissolved by direct methods using SHELXS-97 [2] and refined using alternating cycles of least squares refinements against F^2 (SHELXL-97) [3]. All non H atoms were found in difference Fourier maps and were refined with anisotropic displacement parameters. The H positions were determined by final difference Fourier syntheses. For preparation of the structure drawings the programs DIAMOND [4] and POV-Ray [5] were used.

Further details of the crystal structure investigations have been deposited with the Cambridge Crystallographic Data Center, CCDC 653492. Copies of this information may be obtained free of charge from The Director, CCDC, 12 Union Road, Cambridge, CB2 1EZ, UK (Fax: +44(1223)-336 033; e-mail: fileserv@ccdc.ac.uk or http://www.ccdc.cam.ac.uk).

(*Z*)-3-(4-Methoxyphenyl)-2-(2,3,3-trichloro-1-nitroallylidene)thiazolidin-4-one (12). Yield: 70%, m.p. 176–178 °C; light-brown solid. IR (KBr): $\tilde{\nu}$ 2974, 2925, 1754 (CO), 1606, 1525 (NO_2), 1462, 1365, 1289 (NO_2), 1176, 1024, 962, 917, 882, 814, 759, 720, 684, 621, 561 cm^{-1}. ^1H NMR ($CDCl_3$): δ 7.26–7.10 (m, 2 H, Aryl), 7.04–6.94 (m, 2 H, Aryl), 3.93 (s, 2 H, SCH_2), 3.86 (s, 3 H, OMe) ppm. ^{13}C NMR ($CDCl_3$): δ 173.1 (CO), 161.8 (NCS), 160.9 (*C*OMe), 138.1 (NC_q), 129.3 (CH), 128.2 (CH), 126.3 (CCl), 123.8 (CCl_2), 121.6 (CNO_2), 114.9 (CH), 114.8 (CH), 55.7 (OMe), 31.7 (SCH_2) ppm. MS (EI, 70 eV): *m/z* (%) = 358 (2) $[M – HCl]^+$, 294 (4), 265 (9), 249 (4), 204 (18), 123 (100).

HRMS (ESI): calcd. for $C_{13}H_{10}Cl_3N_2O_4S$ $[M + H]^+$: 394.94214; found: 394.94215.

(*Z*)-3-(4-Ethoxyphenyl)-2-(2,3,3-trichloro-1-nitroallylidene)thiazolidin-4-one (13). Yield: 75%, m.p. 163–165 °C; light-brown solid. IR (KBr): $\tilde{\nu}$ 2985, 2926, 1756 (CO), 1605, 1523 (NO_2), 1473, 1367, 1297 (NO_2), 1177, 1044, 962, 917, 881, 814, 759, 720, 685, 638, 564 cm^{-1}. ^1H NMR ($CDCl_3$): δ 7.23–7.18 (m, 1 H, Aryl), 7.15–7.09 (m, 1 H, Aryl), 7.00–6.94 (m, 2 H, Aryl), 4.12–4.04 (m, 2 H, OCH_2), 3.92 (s, 2 H, SCH_2), 1.44 (t, J = 6.9 Hz, 3 H, Me) ppm. ^{13}C NMR ($CDCl_3$): δ 173.1 (CO), 161.9 (NCS), 160.3 (*C*OCH_2), 138.0 (NC_q), 129.3 (CH), 128.1 (CH), 126.0 (CCl), 123.9 (CCl_2), 120.6 (CNO_2), 115.4 (CH), 115.2 (CH), 63.9 (OCH_2), 31.7 (SCH_2), 14.6 (Me) ppm. MS (EI, 70 eV): *m/z* (%)

= 408 (3) [M^+], 372 (5), 307 (12), 279 (32), 263 (20), 137 (100). HRMS (ESI): calcd. for $C_{14}H_{12}Cl_3N_2O_4S$ [M + H]$^+$: 408.95779; found: 408.95793.

(*Z*)-3-(4-Nitrophenyl)-2-(2,3,3-trichloro-1-nitroallylidene)thiazolidin-4-one (14). Yield: 59%, m.p. 200–202 °C; beige solid. IR (KBr): $\tilde{\nu}$ 2975, 2934, 1740 (CO), 1601, 1526 (NO_2), 1463, 1350, 1289 (NO_2), 1177, 968, 955, 923, 883, 833, 797, 706, 677, 579, 487 cm^{-1}. ^{1}H NMR (DMSO-d$_6$): δ 8.45–8.36 (m, 2 H, Aryl), 7.82–7.74 (m, 2 H, Aryl), 4.21 (d, *J* = 18.8 Hz, 1 H, SCH_2), 4.14 (d, *J* = 18.8 Hz, 1 H, SCH_2) ppm. ^{13}C NMR (DMSO-d$_6$): δ 173.8 (CO), 165.3 (NCS), 148.4 ($C_{Ar}NO_2$), 140.1 (NC_q), 130.8 (CH), 129.6 (CH), 129.3 (CCl), 124.8 (CH), 124.2 (CH), 121.3 (CCl_2), 121.2 (CNO_2), 33.0 (SCH_2) ppm. MS (EI, 70 eV): *m/z* (%) = 373 (16) [M – HCl]$^+$, 327 (30), 298 (16), 280 (29), 264 (100). HRMS (ESI): calcd. for $C_{12}H_7Cl_3N_3O_5S$ [M + H]$^+$: 409.91665; found: 409.91651.

(*Z*)-3-(4-Tolyl)-2-(2,3,3-trichloro-1-nitroallylidene)thiazolidin-4-one (15). Yield: 80%, m.p. 209–210 °C; brown solid. IR (KBr): $\tilde{\nu}$ 2976, 2936, 1743 (CO), 1606, 1518 (NO_2), 1457, 1367, 1292 (NO_2), 1176, 1027, 964, 918, 882, 862, 819, 746, 685, 614, 538, 490 cm^{-1}. ^{1}H NMR ($CDCl_3$): δ 7.34–7.25 (m, 2 H, Aryl), 7.23–7.15 (m, 2 H, Aryl), 3.93 (s, 2 H, SCH_2), 2.41 (s, 3 H, Me) ppm. ^{13}C NMR ($CDCl_3$): δ 172.9 (CO), 161.7 (NCS), 141.1 (NC_q), 131.2 (*C*Me), 130.4 (CH), 130.1 (CH), 129.4 (CCl), 127.8 (CH), 126.6 (CH), 120.5 (CCl_2), 107.0 (CNO_2), 31.8 (SCH_2), 21.3 (Me) ppm. MS (EI, 70 eV): *m/z* (%) = 342 (6) [M – HCl]$^+$, 278 (10), 249 (28), 233 (55), 188 (58), 91 (100). HRMS (ESI): calcd. for $C_{13}H_{10}Cl_3N_2O_3S$ [M + H]$^+$: 378.94722; found: 378.94726.

(*Z*)-3-(2-Methoxyphenyl)-2-(2,3,3-trichloro-1-nitroallylidene)thiazolidin-4-one (16). Yield: 53%, m.p. 177–179 °C; beige solid. IR (KBr): $\tilde{\nu}$ 2967, 2926, 1750 (CO), 1599, 1524 (NO_2), 1462, 1367, 1292 (NO_2), 1184, 1018, 963, 938, 919, 888, 824, 796, 751, 687, 656, 583, 535 cm^{-1}. ^{1}H NMR (DMSO-d$_6$): δ 7.56–7.34 (m, 2 H, Aryl), 7.22–7.01 (m, 2 H, Aryl), 4.30 (d, *J* = 18.7 Hz, 1 H, SCH_2), 4.15 (d, *J* = 18.7 Hz, 1 H, SCH_2), 3.76, 3.75 (s, 3 H, OMe) ppm. ^{13}C NMR (DMSO-d$_6$): δ 173.7, 173.6 (CO), 166.5, 165.2 (NCS), 155.0, 154.4 (*C*OMe), 132.4, 132.3 (CH), 130.9, 128.7 (CH), 130.8 (NC_q), 128.3, 128.2 (CCl), 122.7, 122.1 (CCl_2), 121.6, 121.2 (CNO_2), 120.7, 120.6 (CH), 113.0, 111.6 (CH), 55.8 (OMe), 32.3, 32.1 (SCH_2) ppm. MS (EI, 70 eV): *m/z* (%) = 394 (3) [M^+], 358 (19), 293 (12), 265 (18), 249 (100), 204 (60), 123 (58). HRMS (ESI): calcd. for $C_{13}H_{10}Cl_3N_2O_4S$ [M + H]$^+$: 394.94214; found: 394.94219.

(*Z*)-3-(3-Methoxyphenyl)-2-(2,3,3-trichloro-1-nitroallylidene)thiazolidin-4-one (17). Yield: 55%, m.p .190–192 °C; light-brown solid. IR (KBr): $\tilde{\nu}$ 2969, 2926, 2833, 1758 (CO), 1606, 1520 (NO_2), 1465, 1367, 1289 (NO_2), 1174, 1039, 995, 935, 908, 889, 816, 791, 757, 687, 610, 543 cm^{-1}. ^{1}H NMR (DMSO-d$_6$): δ 7.48–7.37 (m, 1 H, Aryl), 7.14–6.92 (m, 3 H, Aryl), 4.15 (d, *J* = 18.7 Hz, 1 H, SCH_2), 4.10 (d, *J* = 18.7 Hz, 1 H, SCH_2), 3.80, 3.78 (s, 3 H, OMe) ppm. ^{13}C NMR (DMSO-d$_6$): δ 174.0, 173.9

(CO), 165.8, 165.7 (NCS), 159.7, 159.5 (*C*OMe), 135.5, 135.4 (NC_q), 130.3, 129.9 (CH), 128.7, 128.6 (CCl), 121.4 (CNO_2), 121.3 (CCl_2), 121.2, 119.6 (CH), 117.1, 116.5 (CH), 114.4, 112.6 (CH), 55.6, 55.5 (OMe), 32.6, 32.5 (SCH_2) ppm. MS (EI, 70 eV): *m/z* (%) = 394 (6) [M^+], 358 (7), 347 (5), 323 (30), 265 (10), 204 (100). HRMS (ESI): calcd. for $C_{13}H_{10}Cl_3N_2O_4S$ $[M + H]^+$: 394.94214; found: 394.94229.

(*Z*)-3-(2-Tolyl)-2-(2,3,3-trichloro-1-nitroallylidene)thiazolidin-4-one (18). Yield: 70%, m.p. 206–207 °C; beige solid. IR (KBr): $\tilde{\nu}$ 2973, 2915, 1763 (CO), 1595, 1521 (NO_2), 1458, 1361, 1292 (NO_2), 1171, 962, 945, 920, 883, 821, 795, 746, 717, 689, 655, 584, 566 cm^{-1}. ^1H NMR (DMSO-d_6): δ 7.47–7.30 (m, 4 H, Aryl), 4.38 (d, *J* = 18.7 Hz, 0.25 H, SCH_2), 4.35 (d, *J* = 18.7 Hz, 0.75 H, SCH_2), 4.20 (d, *J* = 18.7 Hz, 0.75 H, SCH_2), 4.17 (d, *J* = 18.7 Hz, 0.25 H, SCH_2), 2.15 s (1 H, Me), 2.13 s (2 H, Me) ppm. ^{13}C NMR (DMSO-d_6): δ 173.7, 173.6 (CO), 166.1, 165.1 (NCS), 136.4, 136.3 (*C*Me), 133.7, 133.5 (NC_q), 131.7, 131.0 (CH), 130.9, 130.6 (CH), 129.7, 128.0 (CH), 128.8, 128.5 (CCl), 127.1, 126.9 (CH), 121.3, 121.0 (CCl_2), 121.1, 120.7 (CNO_2), 32.5, 32.4 (SCH_2), 17.8, 17.6 (Me) ppm. MS (EI, 70 eV): *m/z* (%) = 378 (5) [M^+], 342 (14), 278 (19), 249 (14), 233 (100), 188 (72). HRMS (ESI): calcd. for $C_{13}H_{10}Cl_3N_2O_3S$ $[M + H]^+$: 378.94722; found: 378.94728.

(*Z*)-2-(3,3-Dichloro-1-nitro-2-(4-tolylthio)allylidene)-3-*p*-tolylthiazolidin-4-one (**19).** To a stirred suspension of thiazolidin-4-one **15** (0.38 g, 1.00 mmol) and *p*-tolylmercaptane (0.37 g, 3.00 mmol) in methanol (10 mL), sodium methoxide (0.16 g, 3.00 mmol) is added at 0 °C within 10 min. After stirring for 2 h at 0 °C the mixture is kept at rt for 1 d. Then the precipitate is filtered off, washed successively with 5% HCl (20 mL), water (3 × 10 mL), and cold methanol (1 × 5 mL). Final drying afforded pure *p*-tolylthiazolidin-4-one **19** (0.33 g, 70%), m.p. 191–192 °C; orange solid. IR (KBr): $\tilde{\nu}$ 2922, 1749 (CO), 1586, 1522 (NO_2), 1464, 1356, 1281 (NO_2), 1221, 1182, 1025, 956, 916, 889, 861, 823, 791, 742, 688, 620, 540, 510, 447 cm^{-1}. ^1H NMR ($CDCl_3$): δ 7.47 (dd, *J* = 8.1, 2.2 Hz, 1 H, NAr), 7.33 (dd, *J* = 8.1, 2.2 Hz, 1 H, NAr), 7.27 (dd, *J* = 8.1, 2.2 Hz, 1 H, NAr), 7.24 (d, *J* = 8.0 Hz, 2 H, SAr), 7.10 (d, *J* = 8.0 Hz, 2 H, SAr), 7.01 (dd, *J* = 8.1, 2.2 Hz, 1 H, NAr), 3.76 (d, *J* = 18.3 Hz, 1 H, SCH_2), 3.57 (d, *J* = 18.3 Hz, 1 H, SCH_2), 2.42 s (3 H, Me), 2.34 s (3 H, Me) ppm. ^{13}C NMR ($CDCl_3$): δ 173.1 (CO), 160.6 (NCS), 140.5 (C_q), 140.3 (C_q), 134.6 (2CH), 131.7 (*C*Me), 129.8 (2CH), 129.4 (2CH), 128.6 (CH), 126.8 (C_q), 126.4 (CH), 126.2 (C_q), 125.3 (CNO_2), 123.4 (C_q), 32.2 (SCH_2), 21.3 (Me), 21.2 (Me) ppm. MS (EI, 70 eV): *m/z* (%) = 466 (3) [M^+], 431 (3), 399 (6), 342 (35), 278 (28), 249 (30), 91 (100). HRMS (ESI): calcd. for $C_{20}H_{17}Cl_2N_2O_3S_2$ $[M + H]^+$: 467.00522; found: 467.00522.

Methyl 2-(3,4,4-trichloro-1-morpholino-2-nitrobuta-1,3-dienylthio)acetate (20).
To a stirred suspension of acetate **5** (0.34 g, 1.00 mmol) in methanol (5 mL), a solution of morpholine (0.184 g, 2.10 mmol) is added at 0 °C within 5 min. After stirring for 1 h at 0 °C the mixture was left at rt for additional 5 h. Work-up as described for thiazolidinone **19** gave the mercaptoacetate **20** (0.36

g, 93%), m.p. 110–112 °C; yellow solid. IR (KBr): $\tilde{\nu}$ 2970, 2924, 1738 (CO), 1581, 1535 (NO_2), 1459, 1386, 1276 (NO_2), 1201, 1111, 1032, 986, 901, 885, 847, 806, 760, 704, 644, 570, 509 cm^{-1}. ^{1}H NMR ($CDCl_3$): δ 4.40–3.78 (m, 10 H), 3.77 (s, 3 H, OMe) ppm. ^{13}C NMR ($CDCl_3$): δ 168.0 (CO), 166.0 (NCS), 125.8, 125.7 (CCl_2=CCl), 119.0 (CNO_2), 66.3 (OCH_2), 53.8 (NCH_2), 53.3 (OMe), 36.3 (SCH_2) ppm. MS (EI, 70 eV): *m/z* (%) = 390 (2) [M^+], 354 (5), 338 (4), 324 (5), 262 (12), 245 (100). HRMS (ESI): calcd. for $C_{11}H_{14}Cl_3N_2O_5S$ [M + H]$^+$: 390.96835; found: 390.96839.

Ethyl (*E*)-2-(3,4,4-trichloro-1-(naphthalen-1-ylamino)-2-nitrobuta-1,3-dienylthio)acetate (21). In analogy to the morpholine derivative **20**, the aminonaphthalene derivative **21** (0.37 g, 80%) was obtained from acetate **3** (0.36 g, 1.0 mmol) and 1-aminonaphthalene (0.30 g, 2.1 mmol), m.p. 137–138 °C; yellow-green solid. IR (KBr): $\tilde{\nu}$ 2986, 1741 (CO), 1556 (NO_2), 1470, 1360, 1294 (NO_2), 1182, 1149, 1019, 933, 898, 829, 774, 708, 606, 592, 561, 502 cm^{-1}. ^{1}H NMR ($CDCl_3$): δ 12.17 (br s, 1 H, NH), 8.05–7.85 (m, 3 H, Aryl), 7.67–7.48 (m, 4 H, Aryl), 3.98 (q, *J* = 7.2 Hz, 2 H, OCH_2), 2.95 (s, 2 H, SCH_2), 1.12 (t, *J* = 7.2 Hz, 3 H, Me) ppm. ^{13}C NMR ($CDCl_3$): δ 166.6 (CO), 158.3 (NHCS), 134.2 (NHC_q), 133.0 (C_q), 131.3 (C_q), 128.8 (CH), 128.7 (CH), 128.3 (CCl), 127.7 (CH), 127.3 (CH), 125.4 (CH), 123.6 (CCl_2), 122.9 (CH), 122.3 (CNO_2), 121.5 (CH), 62.1 (OCH_2), 34.5 (SCH_2), 13.9 (Me) ppm. MS (EI, 70 eV): *m/z* (%) = 460 (11) [M^+], 416 (15), 379 (8), 343 (14), 143 (100). HRMS (ESI): calcd. for $C_{18}H_{14}Cl_3N_2O_4S$ [M – H]$^-$: 458.97453; found: 458.97478.

General procedure for the preparation of thiazolidinones 22–26.
(2*Z*,5*Z*)-5-Benzylidene-3-phenyl-2-(2,3,3-trichloro-1-nitroallylidene)-thiazolidin-4-one (22). To a suspension of 200 mg (0.547 mmol) of thiazolidinone **7** in 15 mL acetic acid was added 70 mg (0.656 mmol) benzaldehyde and 67 mg (0.656 mmol) triethylamine. The resulting mixture then was refluxed for 5 h. After concentration of this mixture in vacuo to a volume of about 3 mL and cooling to rt, the resulting precipitate was filtered off and consecutively washed with water (2 × 10 mL) and cold methanol (1 × 2 mL). Final drying afforded 199 mg (0.438 mmol, 80%) of thiazolidinone **22** as a dark-yellow solid, m.p. 215-216 °C. IR (KBr): $\tilde{\nu}$ 1716 (CO), 1602, 1593, 1532 (NO_2), 1485, 1363, 1290 (NO_2), 1233, 1162, 935, 722, 690, 554 cm^{-1}. ^{1}H NMR ($CDCl_3$): δ 7.98 (s, 1H), 7.72-7.67 (m, 2H), 7.60-7.49 (m, 6H), 7.41-7.38 (m, 1H), 7.29-7.26 (m, 1H). ^{13}C NMR ($CDCl_3$): δ 166.9 (CO), 155.6 (NCS), 137.9 (CH), 134.1 (C_q), 133.0 (C_q), 131.4 (CH), 131.1 (2CH), 130.7 (CH), 129.95 (CCl), 129.91 (CH), 129.5 (2CH), 129.4 (CH), 128.0 (CH), 127.0 (CH), 122.8 (CNO_2), 120.5 (C_q), 119.8 (C_q) ppm. MS (EI, 70 eV): *m/z* (%) = 452 (7) [M^+], 417 (3), 371 (2), 352 (20), 324 (10), 307 (82), 277 (8), 134 (100). HRMS (ESI): calcd. for $C_{19}H_{11}Cl_3N_2O_3SNa$ [M + Na]$^+$: 474.9454; found: 474.9448.

(2*Z*,5*Z*)-5-Benzylidene-3-(4-chlorophenyl)-2-(2,3,3-trichloro-1-nitroallylidene)thiazolidin-4-one (23). Yield 82%, m.p. 236-238 °C; orange-yellow solid. IR (KBr): $\tilde{\nu}$ 1732 (CO), 1606, 1529 (NO_2), 1489, 1368, 1285 (NO_2), 1220, 1158, 1090, 941, 859, 766, 681, 559 cm^{-1}. ^{1}H NMR ($CDCl_3$): δ 7.98 (s,

1H), 7.72-7.67 (m, 2H), 7.59-7.48 (m, 5H), 7.35-7.31 (m, 1H), 7.25-7.22 (m, 1H). ^{13}C NMR ($CDCl_3$): δ 166.7 (CO), 155.2 (NCS), 138.3 (CH), 136.8 (NC_q), 132.9 (C_q), 132.5 (C_q), 131.5 (CH), 131.1 (2CH), 130.0 (CCl), 129.9 (CH), 129.6 (CH), 129.5 (2CH), 129.4 (CH), 128.4 (CH), 122.8 (CNO_2), 120.4 (C_q), 119.4 (C_q) ppm. MS (EI, 70 eV): *m/z* (%) = 486 (5) [M^+], 451 (3), 405 (2), 386 (12), 357 (7), 341 (30), 134 (100). HRMS (ESI): calcd. for $C_{19}H_{10}Cl_4N_2O_3SNa$ [M + Na]$^+$: 508.9065; found: 508.9058.

(2*Z*,5*Z*)-3-(4-Chlorophenyl)-5-(3,4-dichlorobenzylidene)-2-(2,3,3-trichloro-1-nitroallylidene)thiazolidin-4-one (24). Yield 68%, m.p. 227-229 °C; orange solid. IR (KBr): $\tilde{\nu}$ 1727 (CO), 1604, 1543 (NO_2), 1490, 1365, 1293 (NO_2), 1229, 1172, 1089, 944, 856, 755, 679, 465 cm^{-1}. ^{1}H NMR ($CDCl_3$): δ 7.83 (s, 1H), 7.72 (d, *J* = 2.2 Hz, 1H), 7.64 (d, *J* = 8.4 Hz, 1H), 7.56-7.49 (m, 3H), 7.33-7.30 (m, 1H), 7.24-7.21 (m, 1H). ^{13}C NMR ($CDCl_3$): δ 166.3 (CO), 154.1 (NCS), 137.0 (C_q), 135.7 (C_q), 134.9 (CH), 134.1 (C_q), 132.8 (C_q), 132.7 (CH), 132.3 (C_q), 131.5 (CH), 130.3 (CCl), 130.0 (CH), 129.7 (CH), 129.4 (CH), 129.2 (CH), 128.3 (CH), 123.3 (CNO_2), 121.6 (C_q), 120.1 (C_q) ppm. MS (EI, 70 eV): *m/z* (%) = 554 (6) [M^+], 519 (3), 456 (22), 442 (4), 409 (55), 202 (100). HRMS (ESI): calcd. for $C_{19}H_8Cl_6N_2O_3S$ [M]$^+$: 553.8387; found: 553.8371.

(2Z,5*Z*)-5-(3,4-Dichlorobenzylidene)-3-(4-fluorophenyl)-2-(2,3,3-trichloro-1-nitroallylidene)thiazolidin-4-one (25). Yield 82%, m.p. 217-219 °C; dark-yellow solid. IR (KBr): $\tilde{\nu}$ 1718 (CO), 1602, 1537 (NO_2), 1471, 1394, 1294 (NO_2), 1225, 1164, 1030, 987, 918, 818, 753, 683, 555 cm^{-1}. ^{1}H NMR ($CDCl_3$): δ 7.83 (s, 1H), 7.72 (d, *J* = 2.1 Hz, 1H), 7.64 (d, *J* = 8.4 Hz, 1H), 7.54 (dd, *J* = 8.4, 2.1 Hz, 1H), 7.40-7.35 (m, 1H), 7.31-7.21 (m, 3H). ^{13}C NMR ($CDCl_3$): δ 166.5 (CO), 163.5 (CF, $J_{C,F}$ = 252.3 Hz), 154.3 (NCS), 135.7 (C_q), 134.8 (CH), 134.0 (C_q), 132.8 (C_q), 132.6 (CH), 131.5 (CH), 130.2 (CCl), 130.1 (CH, $J_{C,F}$ = 9.4 Hz), 129.7 (NC_q, $J_{C,F}$ = 3.4 Hz), 129.2 (CH), 129.1 (CH, $J_{C,F}$ = 9.0 Hz), 123.2 (CNO_2), 121.6 (C_q), 120.2 (C_q), 117.0 (CH, $J_{C,F}$ = 22.7 Hz), 116.7 (CH, $J_{C,F}$ = 23.6 Hz) ppm. MS (EI, 70 eV): *m/z* (%) = 538 (3) [M^+], 503 (2), 457 (5), 440 (8), 393 (21), 202 (100). HRMS (ESI): calcd. for $C_{19}H_9Cl_5FN_2O_3S$ [M + H]$^+$: 538.87605; found: 538.8764.

(2*Z*,5*Z*)-5-Benzylidene-3-(4-methoxyphenyl)-2-(2,3,3-trichloro-1-nitroallylidene)thiazolidin-4-one (26). Yield 94%, m.p. 193-194 °C; yellow solid. IR (KBr): $\tilde{\nu}$ 2927, 1730 (CO), 1603, 1531 (NO_2), 1511, 1366, 1285 (NO_2), 1255, 1167, 1030, 938, 824, 764, 685, 550 cm^{-1}. ^{1}H NMR ($CDCl_3$): δ 7.97 (s, 1H), 7.72-7.68 (m, 2H), 7.58-7.48 (m, 3H), 7.32-7.28 (m, 1H), 7.21-7.17 (m, 1H), 7.05-6.98 (m, 2H), 3.88 (s, 3H, OMe). ^{13}C NMR ($CDCl_3$): δ 167.2 (CO), 160.9 (C_q), 155.8 (NCS), 137.9 (CH), 133.0 (C_q), 131.3 (CH), 131.1 (2CH), 129.52 (CCl), 129.50 (2CH), 129.4 (CH), 128.3 (CH), 126.5 (C_q), 122.7 (CNO_2), 120.6 (C_q), 119.8 (C_q), 114.9 (CH), 114.8 (CH), 55.7 (OMe) ppm. MS (EI, 70 eV): *m/z* (%) = 482 (3) [M^+], 447 (2), 401 (1), 382 (7), 353 (9), 337 (25), 134 (78), 123 (100). HRMS (ESI): calcd. for $C_{20}H_{14}Cl_3N_2O_4S$ [M + H]$^+$: 482.97399; found: 482.9734.

3-Hydrazonomethyl-4-nitro-5-(4-chlorophenylamino)-1*H*-pyrazole (27). To a stirred suspension of thiazolidin-4-one **8** (0.40 g, 1.00 mmol) in methanol (10 mL) at 0–5 °C was added a solution of hydrazine hydrate (0.25 g, 5.00 mmol) in methanol (5 mL) dropwise within 5 min. After 1 h with stirring at 5 °C and 3 h at rt, the precipitate was filtered off, washed with water (15 mL) and cold methanol (10 mL). After drying pyrazole **27** was obtained (0.18 g, 64%), m.p. 238–240 °C; orange solid. IR (KBr): $\tilde{\nu}$ 3371, 1599, 1575 (NO_2), 1465, 1364 (NO_2), 1290, 1238, 1173, 1093, 903, 821, 767, 608, 502 cm^{-1}. ^1H NMR (DMSO-d_6): δ 13.31 (br s, 1 H, NH), 8.73 (br s, 1 H, NH), 8.18 (br s, 2 H, NH_2), 8.07 (s, 1 H, CH), 7.75 (d, J = 8.6 Hz, 2 H, Aryl), 7.31 (d, J = 8.6 Hz, 2 H, Aryl) ppm. ^{13}C NMR (DMSO-d_6): δ 146.9 (C_5), 139.6, 138.9, 128.7 (2CH, Aryl), 124.7 (CCl), 122.6 (CH), 119.5 (2CH, Aryl), 117.6 (CNO_2) ppm. MS (EI, 70 eV): *m/z* (%) = 280 (100) [M^+], 252 (3), 189 (15), 154 (6), 111 (22). HRMS (ESI): calcd. for $C_{10}H_{10}ClN_6O_2$ $[M + H]^+$: 281.05483; found: 281.05484.

3-Hydrazonomethyl-4-nitro-5-(4-fluorophenylamino)-1*H*-pyrazole (28) was prepared from **25** and hydrazine hydrate in analogy to the synthesis of **27**. Yield 52%, m.p. 210-211 °C; purple solid. IR (KBr): $\tilde{\nu}$ 3382, 3222, 1597, 1596 (NO_2), 1509, 1456, 1366 (NO_2), 1286, 1220, 1157, 1109, 896, 822, 767, 678, 508 cm-1. ^1H NMR (DMSO-d_6): δ 13.31 (br s, 1 H, NH), 8.65 (br s, 1 H, NH), 8.17 (br s, 2 H, NH_2), 8.07 (s, 1 H, CH), 7.74 (dd, J = 9.1, 4.6 Hz, 2 H, Aryl), 7.13 (dd, J = 9.1, 8.6 Hz, 2 H, Aryl) ppm. ^{13}C NMR (DMSO-d): δ 157.1 (CF, $J_{C,F}$ = 237.5 Hz), 147.3 (C5), 138.8, 137.1 (NCq, $J_{C,F}$ = 2.2 Hz), 122.7 (CH), 119.6 (CH, $J_{C,F}$ = 7.3 Hz), 117.4 (CNO_2), 115.4 (CH, $J_{C,F}$ = 22.3 Hz) ppm. MS (EI, 70 eV): *m/z* (%) = 264 (78) [M^+], 217 (6), 173 (15), 120 (53), 105 (100). HRMS (ESI): calcd. for $C_{10}H_{10}FN_6O_2$ $[M + H]^+$: 265.08493; found: 265.08490.

Ethyl 2-((1,3,4,4-tetrachloro-2-nitrobuta-1,3-dien-1-yl)thio)propanoate (29) was obtained in analogy to **3** from nitrodiene **1** and 2.2 equiv of ethyl 2-mercaptopropanoate as a mixture of *Z*- and *E*-isomers in a 4:1 ratio. Yield 78%, yellow oil. IR (NaCl): $\tilde{\nu}$ 2984, 2938, 1731 (C=O), 1603, 1532 (NO_2), 1451, 1366, 1310 (NO_2), 1176, 1079, 1010, 945, 920, 859, 829, 761, 688 cm^{-1}. ^1H NMR ($CDCl_3$): δ 4.32 (q, J = 7.4 Hz, 1.25 H, SCH), 4.24 (q, J = 7.1 Hz, 2.5 H, OCH_2), 1.66 (d, J = 7.4 Hz, 3 H, $CHCH_3$), 1.64 (d, J = 7.4 Hz, 0.75 H, $CHCH_3$), 1.30 (t, J = 7.1 Hz, 0.75 H, CH_3), 1.29 (t, J = 7.1 Hz, 3 H, CH_3) ppm. ^{13}C NMR ($CDCl_3$) major isomer: δ 170.4 (CO), 155.2 (Cl-C-S), 139.1 (CNO_2), 129.1 (CCl), 120.9 (CCl_2), 62.5 (OCH_2), 47.0 (SCH), 16.9 ($CHCH_3$), 14.0 (CH_3) ppm. ^{13}C NMR ($CDCl_3$) minor isomer: δ 170.1 (CO), 130.3 (CCl), 120.2 (CCl_2), 61.4 (OCH_2), 45.0 (SCH), 17.0 ($CHCH_3$), 14.1 (CH_3) ppm. The carbon atoms of the nitrovinylic group could not be detected. MS (EI, 70 eV): *m/z* (%) = 367 (1) [M^+], 332 (5), 322 (2), 266 (100). HRMS (ESI): calcd. for $C_9H_8Cl_4NO_4S$ $[M – H]^+$: 365.89336; found 365.8934.

Thiazolidinones **30–32** were synthetized each as a mixture of isomers following the general procedure that was described for the preparation of **7–18.**

3-(4-Chlorophenyl)-5-methyl-2-(2,3,3-trichloro-1-nitroallylidene)thiazolidin-4-one (30). Isomeric ratio: 2:1. Total yield: 47%, m.p. 170–171 °C; yellow solid. IR (KBr): $\tilde{\nu}$ 2928, 1750 (CO), 1595, 1527 (NO_2), 1485, 1403, 1357, 1295 (NO_2), 1175, 1086, 992, 943, 903, 817, 786, 727, 684, 552, 469 cm^{-1}. ^1H NMR ($CDCl_3$): δ 7.51–7.45 (m, 2 H, Aryl), 7.27-7.13 (m, 2 H, Aryl), 4.09 (q, J = 7.3 Hz, 0.67 H, SCH), 4.06 (q, J = 7.3 Hz, 0.33 H, SCH), 1.76 (d, J = 7.3 Hz, 2 H, Me), 1.75 (d, J = 7.3 Hz, 1 H, Me) ppm. ^{13}C NMR ($CDCl_3$): major isomer δ 176.0 (CO), 159.8 (NCS), 136.7 (NC_q), 132.6 ($C_{q,aryl}Cl$), 130.0 (CH), 129.7 (CH), 129.6 (CCl), 129.2 (CH), 128.3 (CH), 120.7 (CCl_2), 120.6 (CNO_2), 40.2 (SCH), 18.1 (Me) ppm. ^{13}C NMR ($CDCl_3$): minor isomer δ 176.02 (CO), 159.9 (NCS), 136.8 (NC_q), 132.5 ($C_{q,aryl}Cl$), 129.9 (CH), 129.8 (CCl), 129.5 (CH), 129.3 (CH), 128.2 (CH), 40.4 (SCH), 18.8 (Me) ppm. Two carbon atoms of allylic group could not be detected. MS (EI, 70 eV): *m/z* (%) = 412 (7) [M^+], 377 (20), 331 (7), 312 (20), 284 (25), 267 (100), 153 (33). HRMS (ESI): calcd. for $C_{13}H_9Cl_4N_2O_3S$ [M + H]$^+$: 412.90880; found: 412.9083.

3-(4-Methoxyphenyl)-5-methyl-2-(2,3,3-trichloro-1-nitroallylidene)-thiazolidin-4-one (31). Isomeric ratio: 3:1. Total yield: 54%, m.p. 146–147 °C; light-brown solid. IR (KBr): $\tilde{\nu}$ 2927, 1753 (CO), 1604, 1524 (NO_2), 1463, 1361, 1295 (NO_2), 1249, 1169, 1035, 991, 945, 900, 819, 738, 686, 592, 509 cm^{-1}. ^1H NMR ($CDCl_3$): δ 7.25–7.08 (m, 2 H, Aryl), 7.01-6.95 (m, 2 H, Aryl), 4.06 (q, J = 7.3 Hz, 0.67 H, SCH), 4.04 (q, J = 7.3 Hz, 0.33 H, SCH), 3.85 (s, 3H, OMe), 1.76 (d, J = 7.3 Hz, 1 H, Me), 1.74 (d, J = 7.3 Hz, 2 H, Me) ppm. ^{13}C NMR ($CDCl_3$): major isomer δ 176.4 (CO), 160.9 (C-O), 160.5 (NCS), 129.1 (CH), 128.2 (CH), 126.6 (NC_q), 123.6 (CCl), 120.9 (CCl_2), 119.2 (CNO_2), 115.0 (CH), 114.80 (CH), 55.7 (OMe), 40.1 (SCH), 18.1 (Me) ppm. ^{13}C NMR ($CDCl_3$): minor isomer δ 176.6 (CO), 160.8 (C-O), 160.7 (NCS), 129.3 (CH), 128.1 (CH), 126.5 (NC_q), 125.1 (CCl), 120.8 (CCl_2), 114.81 (CH), 114.82 (CH), 55.7 (OMe), 40.2 (SCH), 18.8 (Me) ppm. The carbon adjacent to the nitro group could not be detected. MS (EI, 70 eV): *m/z* (%) = 408 (2) [M^+], 373 (3), 308 (4), 301 (2), 279 (11), 149 (12), 123 (100). HRMS (ESI): calcd. for $C_{14}H_{12}Cl_3N_2O_4S$ [M + H]$^+$: 408.95834; found: 408.9578.

5-Methyl-3-(4-tolyl)-2-(2,3,3-trichloro-1-nitroallylidene)thiazolidin-4-one (32). Isomeric ratio 9:1. Total yield: 48%, m.p. 185–186 °C; yellow solid. IR (KBr): $\tilde{\nu}$ 2928, 1753 (CO), 1597, 1525 (NO_2), 1463, 1360, 1295 (NO_2), 1218, 1176, 992, 940, 902, 818, 727, 687, 583, 540 cm^{-1}. ^1H NMR ($CDCl_3$): δ 7.29 (d, J = 8.2 Hz, 2 H, Aryl), 7.21-7.16 (m, 1 H, Aryl), 7.11-7.04 (m, 1 H, Aryl), 4.07 (q, J = 7.3 Hz, 1 H, SCH), 2.41 (s, 3 H, Me), 1.76 (d, J = 7.3 Hz, 0.3 H, Me), 1.75 (d, J = 7.3 Hz, 2.7 H, Me) ppm. ^{13}C NMR ($CDCl_3$): major isomer δ 176.3 (CO), 160.4 (NCS), 140.9 (Me*C*), 131.6 (NC_q), 130.3 (CH), 130.1 (CH), 129.2 (CCl), 127.6 (CH), 126.6 (CH), 123.6 (CNO_2), 120.8 (CCl_2), 40.2 (SCH), 21.3 (Me), 18.2 (Me) ppm. For the minor isomer only the CH carbon atoms were detected at 130.2,

130.0, 127.8, 126.5, 40.4, and 18.9 ppm. MS (EI, 70 eV): *m/z* (%) = 392 (5) [M^+], 357 (15), 311 (2), 292 (12), 263 (36), 247 (100), 133 (10). HRMS (ESI): calcd. for $C_{14}H_{12}Cl_3N_2O_3S$ [M + H]$^+$: 392.96342; found: 392.9637.

2-(3,3-Dichloro-2-((4-chlorophenyl)thio)-1-nitroallylidene)-5-methyl-3-(*p*-tolyl)thiazolidin-4-one (33). To a stirred suspension of thiazolidin-4-one **32** (0.39 g, 1.00 mmol) and 4-chlorothiophenol (0.29 g, 2.00 mmol) in ethanol (10 mL), sodium ethoxide (0.14 g, 2.00 mmol) was added at 0 °C within 10 min. After 1 h at 0 °C the mixture was stirred at 40–45 °C for 1 d. The resulting precipitate was filtered off, washed successively with 5% HCl (20 mL), water (3 × 10 mL), and cold methanol (1 × 5 mL). Final drying in vacuo afforded thiazolidinone **33** (0.30 g, 60%) as a yellow solid. Isomeric ratio 2:1, m.p. 197–198 °C. IR (KBr): $\tilde{\nu}$ 3079, 2923, 1742 (CO), 1589, 1524 (NO_2), 1462, 1349, 1284 (NO_2), 1220, 1190, 1022, 988, 900, 837, 822, 688, 530, 510, 493 cm^{-1}. ^{1}H NMR ($CDCl_3$): δ 7.40 (dd, J = 8.1, 2.2 Hz, 0.5 H), 7.35-7.23 (m, 10 H), 7.05 (d, J = 8.2 Hz, 1 H), 7.01 (dd, J = 8.1, 2.2 Hz, 0.5 H), 3.93 (q, J = 7.2 Hz, 1 H, SCH), 3.79 (q, J = 7.2 Hz, 0.5 H, SCH), 2.42 (s, 4.5 H, Me), 1.66 (d, J = 7.2 Hz, 1.5 H, Me), 1.61 (d, J = 7.2 Hz, 3 H, Me) ppm. ^{13}C NMR ($CDCl_3$): major isomer δ 176.4 (CO), 159.6 (NCS), 140.6 (C_q), 135.8 (C_q), 135.0 (2CH), 131.9 (C_q), 129.7 (2CH), 129.4 (2CH), 128.6 (C_q), 128.4 (CH), 126.6 (CH), 126.0 (C_q), 125.7 (C_q), 124.7 (CNO_2), 40.1 (SCH), 21.3 (Me), 18.5 (Me) ppm. Minor isomer δ 176.5 (CO), 159.5 (NCS), 140.5 (C_q), 135.9 (C_q), 135.1 (2CH), 132.1 (C_q), 129.6 (2CH), 129.3 (2CH), 128.7 (C_q), 128.3 (CH), 126.4 (CH), 126.2 (C_q), 125.6 (C_q), 125.0 (CNO_2), 40.6 (SCH), 21.2 (Me), 18.0 (Me) ppm. MS (EI, 70 eV): *m/z* (%) = 500 (2) [M^+], 465 (5), 434 (9), 409 (10), 357 (80), 263 (93), 91 (100). HRMS (ESI): calcd. for $C_{20}H_{16}Cl_3N_2O_3S_2$ [M + H]$^+$: 500.96679; found: 500.9662.

References

1. Potkin, V. I.; Zapol'skii, V. A.; Kaberdin, R. V. *Izv. Akad. Nauk Bel., Ser. Khim. Nauk.* **1996**, *40*, 68–71.

2. Sheldrick, G. M., SHELXS-97, Program for the Solution of Crystal Structures, University of Göttingen, Göttingen (Germany) **1997**. See also: Sheldrick, G.M. *Acta Crystallogr. Sect. A* **1990**, *A46*, 467–473. doi: 10.1107/S0108767390000277.

3. Sheldrick, G. M., SHELXL–97, Program for the Refinement of Crystal Structures, University of Göttingen, Göttingen (Germany) 1997. See also: Sheldrick, G.M. *Acta Crystallogr. Sect. A* **2008**, *A64*, 112–122. doi: 10.1107/S0108767307043930.

4. Brandenburg, K., DIAMOND (version 3.2g), Crystal and Molecular Structure Visualization, Crystal Impact – K. Brandenburg & H. Putz GbR, Bonn (Germany) 2011. See also: http://www.crystalimpact.com/diamond/.

5. POV-RAY (version 3.6), Trademark of Persistence of Vision Raytracer Pty. Ltd., Williamstown, Victoria (Australia); Copyright Hallam Oaks Pty. Ltd., 2005. http://povray.org/.
